# Kibi Plateau: A stable-coherent tectonic unit in the active Japanese Islands

**DOI:** 10.1038/s41598-020-60448-x

**Published:** 2020-03-02

**Authors:** Takafumi Sonehara, Koshi Yagi, Hiroyuki Takeshita, Kazumasa Aoki, Shogo Aoki, Yo-ichiro Otofuji, Tetsumaru Itaya

**Affiliations:** 1 Hiruzen Institute for Geology and Chronology, 2-5 Nakashima, Naka-ku, Okayama, 703-8252 Japan; 2 Graduate school of Science, Okayama University of Science, 1-1 Ridai-cho, Kita-ku, Okayama, 700-0005 Japan; 3 Faculty of Biosphere-Geosphere Science, Okayama University of Science, 1-1 Ridai-cho, Kita-ku, Okayama, 700-0005 Japan; 4 Institute of GeoHistory, Japan Geochronology Network, 1599 Susai, Akaiwa, 701-2503 Japan; 5 Engineering Geology Center, Hiruzen Institute for Geology and Chronology, 2-12 Nakashima, Naka-ku, Okayama, 703-8252 Japan; 6 Institute of Frontier Science and Technology, Okayama University of Science, 1-1 Ridai-cho, Kita-ku, Okayama, 700-0005 Japan

**Keywords:** Stratigraphy, Geomorphology, Tectonics

## Abstract

The Kibi Plateau in the active Japanese Islands consists of mainly Permian to Cretaceous rocks that have been deeply weathered into a red soil, comprising a peneplain with U-shaped valley. Systematic geological analyses of the Eocene fluvial deposits revealed the paleo-rivers that existed in the eastern Asian continent and streamed out to the paleo-Pacific Ocean. Each paleo-river is traced in a flow line shape without any significant vertical and horizontal displacement. The Eocene shallow marine sediments in a possible coastal region have no relevant inclination. These geological data strongly suggest that the Kibi Plateau has been a stable-coherent tectonic unit since the Eocene through the opening of the Japan Sea and the associated quick rotation of SW Japan in the Middle Miocene. The Kibi Plateau region with a thick crust over 30 km existed as a stable eastern segment of the Asian continent in the Eocene. The Kibi Plateau tectonic unit drifted to the south without any destruction due to the peripheral successive tectonic events such as the Philippine Sea plate subduction and the reactivation of Median Tectonic Line. No subduction related arc volcanism since the Eocene has also influenced to preserve the stable tectonic unit.

## Introduction

The Japanese Islands and surrounding areas consist of four plates (i.e., the Pacific, Eurasian, Philippine Sea and North American plates) with two triple junctions and have experienced active subductions. These subductions have caused intense seismic and volcanic activities, thus indicating that the region is one of the most dangerous areas for natural disasters in the world. However, the Kibi Plateau in SW Japan consists of mainly Permian to Cretaceous rocks that have been deeply weathered into a red soil, comprising a peneplain, which was discovered in 1908^1–3^. Recently, Eocene fluvial deposits have been found in the Kibi Plateau region^4–6^. Systematic geological analyses of these Eocene fluvial deposits revealed the paleo-rivers that existed in the eastern Asian continent and streamed out to the paleo-Pacific Ocean. The Eocene shallow marine sediments in a possible coastal region have no relevant inclination. An important point is that each paleo-river is traced in a flow line shape without any significant vertical and horizontal displacement and the marine sediments have no significant inclination, thus suggesting that the Kibi Plateau has been a stable-coherent tectonic unit since the Eocene through the opening of the Japan Sea and the associated quick rotation (an angular velocity of 20°/Myr) of SW Japan that took place in the Middle Miocene^7,8^. Its average uplift rate is estimated to be ca. 0.002 mm/y since the Eocene. The seismic tomography of SW Japan^9,10^ also supports its future stability. Here, we report the geological data revealing a stable-coherent tectonic unit in the active Japanese Islands, whose area is ca. four times larger than the area of Tokyo (ca. 1,787 km^2^) and discuss the reason why the Kibi Plateau tectonic unit drifted to the south without any destruction and preserved the stable-coherent tectonic unit.

## Geological Data

**Figure 1:**
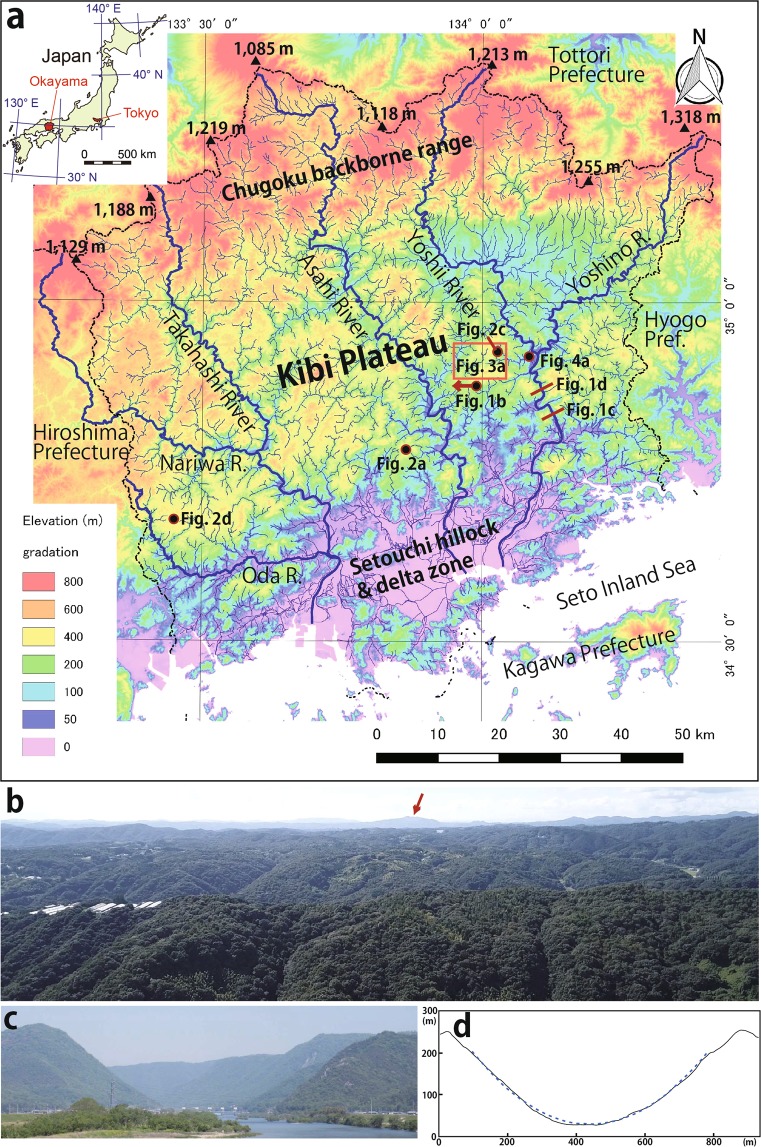
(**a**) Topographic map of Okayama Prefecture and the surrounding area in SW Japan. The area has been divided topographically into three zones, (1) Chugoku backbone range, where the main peaks are higher than 1,000 m, (2) Kibi Plateau consisting of a low-relief surface (200–600 m) and inselberg and (3) Setouchi hillock and delta zone of the three main rivers of the Takahashi, Asahi and Yoshii. (**b**) Photo of the Kibi Plateau taken from the point (**b**: the elevation is ca. 300 m) marked in (**a**) to the westward, showing the low-relief surface (200–600 m) and the inselberg. The low-relief surface slopes down gently to the south from the north. The distance to the inselberg (Tenjin Mt. of 777 m height that is highest in the Kibi Plateau) with an arrow from the point (**b**) is 54 km. The Jinseki Plateau in the eastern part of Hiroshima Prefecture behind Tenjin Mt. is visible. It is ca. 70 km far from the view point. (**c**) Photo of the U-shaped valley of the Yoshii river, taken from the point (**c**) marked in (**a**) to the northward. The width of the Yoshii river at the point is 220 m. Difference in elevation between the Yoshii river and the left-hand side peak is 390 m. (**d**) The cross-section of the Yoshi river at the location (**d**) shown in (**a**). It shows the form of the “U” and is accurately approximated by a parabola (The blue dotted line shows Y = a X^2^).

**Figure 2:**
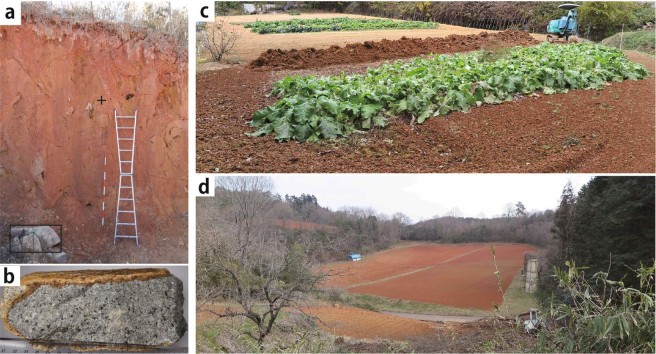
(**a**) Photo of the deeply weathered rocks changing into red soil. The location is the site (**a**) shown in (**a**), where the red soil has been exploited for use as an impermeable material for the local farm pond. The sample for the major element analyses was collected from the “+” marked point. Its host rock is observed as the core stone existing at the lower left in this photo. The red and white pole, as a scale, is 2 m in length. (**b**) Photo of the host rock sample (Cretaceous biotite granite) for the major element analyses collected from the core stone at the lower left in the photo (**a**). (**c**) Photo taken in the burdock harvest at the burdock farm in Koresato village (the elevation of ca. 300 m) situated at the northern part of Akaiwa city located in the Eastern part of Okayama Prefecture. The yellow colored farm behind the burdock farm lies fallow. (**d**) Photo taken before planting burdock seeds at the burdock farm in Meiji-Gonbo village (The official name is Yoshii, where the elevation is ca. 400 m), which is situated at the northern part of Ibara city located in the western part of Okayama Prefecture.

**Figure 3:**
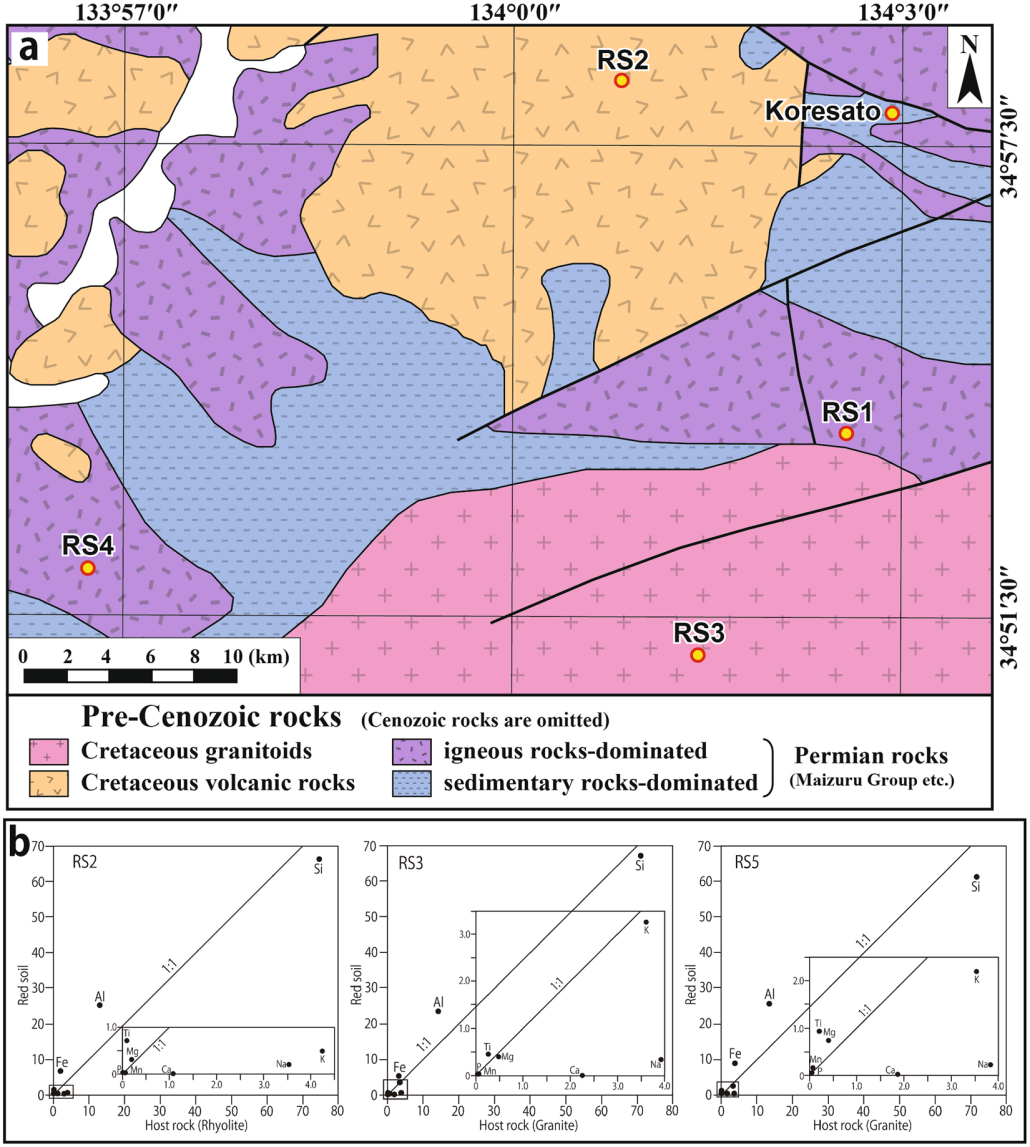
(**a**) Geological map (simplified from ref. ^11^) in the quadrilateral area (**a**) marked in Fig. 1a. The figure shows the locations of the samples for the major element analyses collected from the four sites of pelite (RS1) and schistose rock (RS4) from the Permian Maizuru Group, and Cretaceous rhyolite (RS2) and Cretaceous granite (RS3) in the northern part of Akaiwa city. (**b**) The isocon diagrams^14^ based on the major element composition data of the deeply weathered rocks and their host rocks. The chemical composition data are shown in Table 1. Si: SiO_2_, Ti: TiO_2_, Al: Al_2_O_3_, Fe: Fe_2_O_3_, Mn: MnO, Mg: MgO, Ca: CaO, Na: Na_2_O, K: K_2_O, P: P_2_O_5_.

The Kibi Plateau is situated mainly in the central part of Okayama Prefecture in SW Japan (Fig. 1a) and is a peneplain^1–3^ that consists of a low-relief surface (200–600 m) and an inselberg (Fig. 1b). Its lithology is composed of mainly Permian sedimentary and igneous rocks (i.e., the Maizuru Group including Yakuno ophiolites and the Ultra-Tamba Group), Triassic sedimentary and metamorphosed rocks (i.e., the Nariwa Group and Suo belt), a Jurassic accretionary complex (i.e., the Mino-Tamba Group), Cretaceous rhyolites and granitoids, and minor Cenozoic sedimentary rocks^11^. Three major rivers (i.e., the Takahashi, Asahi and Yoshii) flowing from north to south have deeply eroded the lithology, creating a steep-walled valley with a U-shape (Fig. 1c,d). However, the steepness of the wall depends on the lithology, e.g., being lower for Cretaceous granitoids than rhyolites and intermediate for Permian sedimentary rocks of the Maizuru Group^12^. The uplift rate of the Kibi Plateau is the smallest in Japan, being 0.03 mm/y since the Middle Miocene as estimated by ref. ^13^. The average rates since the Eocene may be much lower. Deeply weathered rocks are commonly observed at the low-relief surface of the Permian to Cretaceous lithology and are transitioning into red soil. Figure 2a shows the red soil exploitation site used as an impermeable material for the local farm pond. This type of red soil is always observed at the low-relief surface in the Kibi Plateau beneath the vegetation surface. This red soil has also been used as an agricultural soil, specifically in the burdock farms, such as those in Koresato and Meiji-Gonbo villages. The former (Fig. 2c: the elevation of ca. 300 m) is situated at the northern part of Akaiwa city located in the Eastern part of Okayama Prefecture. The later (Fig. 2d: The official name is Yoshii, where the elevation is ca. 400 m) is situated at the northern part of Ibara city located in the western part of Okayama Prefecture. The major elemental analyses of the weathered rock (Fig. 2a) and its host rock (Cretaceous granitic rock) occurring as a core stone (Fig. 2b) were carried out to observe the chemical weathering process. The results together with four additional sample data points (Fig. 3a) are shown in Table 1. The isocon diagrams (Fig. 3b)^14^ are based on the major element composition data of the deeply weathered rocks and the host rocks. The diagrams show that the weathering experienced aluminum and iron enrichment processes in which Na_2_O, K_2_O, CaO and SiO_2_ were depleted in comparison with the host rock. These red soil formations are chemically similar to the laterite formation observed in the stable continental lithology.

**Table 1:** Major elemental compositions of the deeply weathered rocks transitioning into the red soil and their host rocks.

Site	RS1	RS2		RS3		RS4	RS5	
(wt%)	Red soil	Red soil	Host rock	Red soil	Host rock	Red soil	Red soil	Host rock
SiO_2_	54.20	65.95	75.02	66.92	71.27	79.22	61.08	71.78
TiO_2_	1.51	0.70	0.10	0.44	0.27	0.50	0.91	0.24
Al_2_O_3_	29.03	25.16	13.19	23.09	14.44	14.56	25.40	13.58
ΣFe_2_O_3_	12.41	6.57	2.05	5.13	3.40	3.59	8.67	4.17
MnO	0.04	0.01	0.05	0.02	0.06	0.02	0.16	0.09
MgO	0.76	0.30	0.20	0.38	0.49	0.28	0.72	0.42
CaO	0.00	0.00	1.10	0.00	2.27	0.00	0.00	1.89
Na_2_O	0.19	0.20	3.55	0.32	3.96	0.19	0.21	3.84
K_2_O	1.07	0.48	4.26	3.26	3.63	0.61	2.18	3.55
P_2_O_5_	0.09	0.01	0.01	0.02	0.07	0.01	0.04	0.07
Total	99.30	99.38	99.53	99.58	99.86	98.98	99.37	99.63
LOI	14.85	12.49	0.63	8.26	0.24	6.30	13.02	0.66
**Host rock type**	**Schistose rock**	**Rhyolite**		**Granite**		**Pelite**	**Granite**	
**Age**	**Permian**	**Cretaceous**		**Cretaceous**		**Permian**	**Cretaceous**	
Latitude	N34°53'38.7"	N34°55'54.6"		N34°52'15.2"	N34°52'19.1"	N34°52'48.1"	N34°47'24.3"	
Longitude	E134°02'34.8"	E134°00'47.6"		E134°01'26.6"	E134°01'26.0"	E133°56'44.0"	E133°51'54.6"	
Elevation	360 m	422 m		250 m	240 m	288 m	308 m	

The sampling sites of RS1 to RS4 are shown in Fig. 3a and the site (Fig. 2a) of RS5 is shown in Fig. 1a. This table also shows the host rock types and their ages, and the latitude, longitude and elevation of the sampling sites.

**Figure 4:**
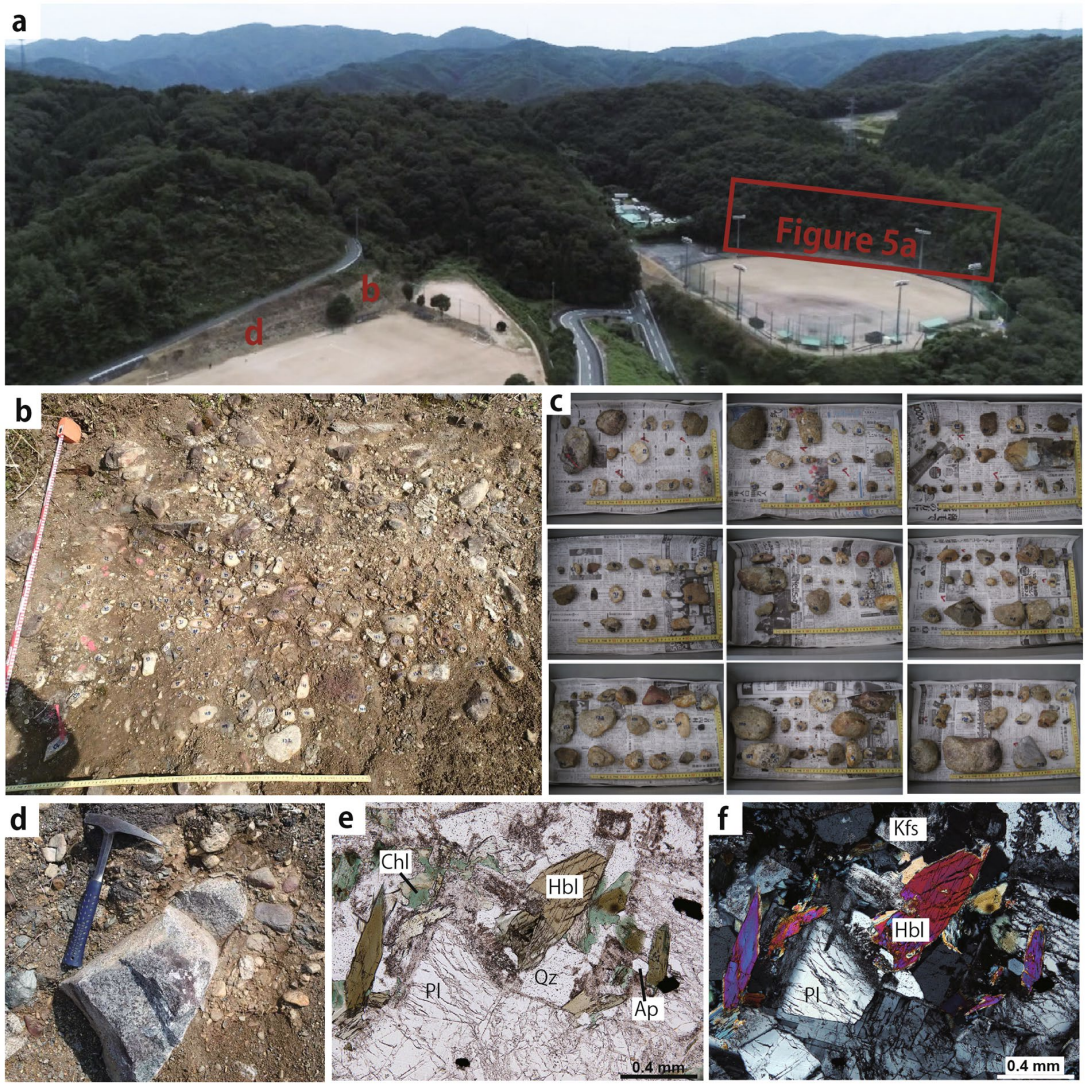
(**a**) Photo of the developed land used to make the large sport square in Susai town of Akaiwa city, situated at the eastern part of the Kibi Plateau; its location is the site (**a**) shown in Fig. 1a. The outcrop of the Eocene fluvial deposit (conglomerate here) appeared during the development. The squared area in red was sketched to show the occurrence of the tuff. (**b**) Photo showing the site used to analyze the rock types of the gravels. The width of the photo is ca. 1.5 m. (**c**) The gravels (174 samples) collected from the area (**b**). The width of each photo is ca. 50 cm. The gravels are variable in their size, shape (angular to rounded gravel) and rock type. (**d**) Photo showing the granodiorite gravel that was dated by the K-Ar method. The pick hammer by the granodiorite is ca. 40 cm in length. (**e**,**f**) Photomicrographs of the granodiorite. Photo e is under plane polarized light, and photo f is under crossed polarized light. The sample contains fresh hornblende and plagioclase without any alterations. Hbl: hornblende, Pl: plagioclase, Kfs: K-feldspar, Qz: quartz, Ap: apatite, Chl: chlorite.

**Figure 5:**
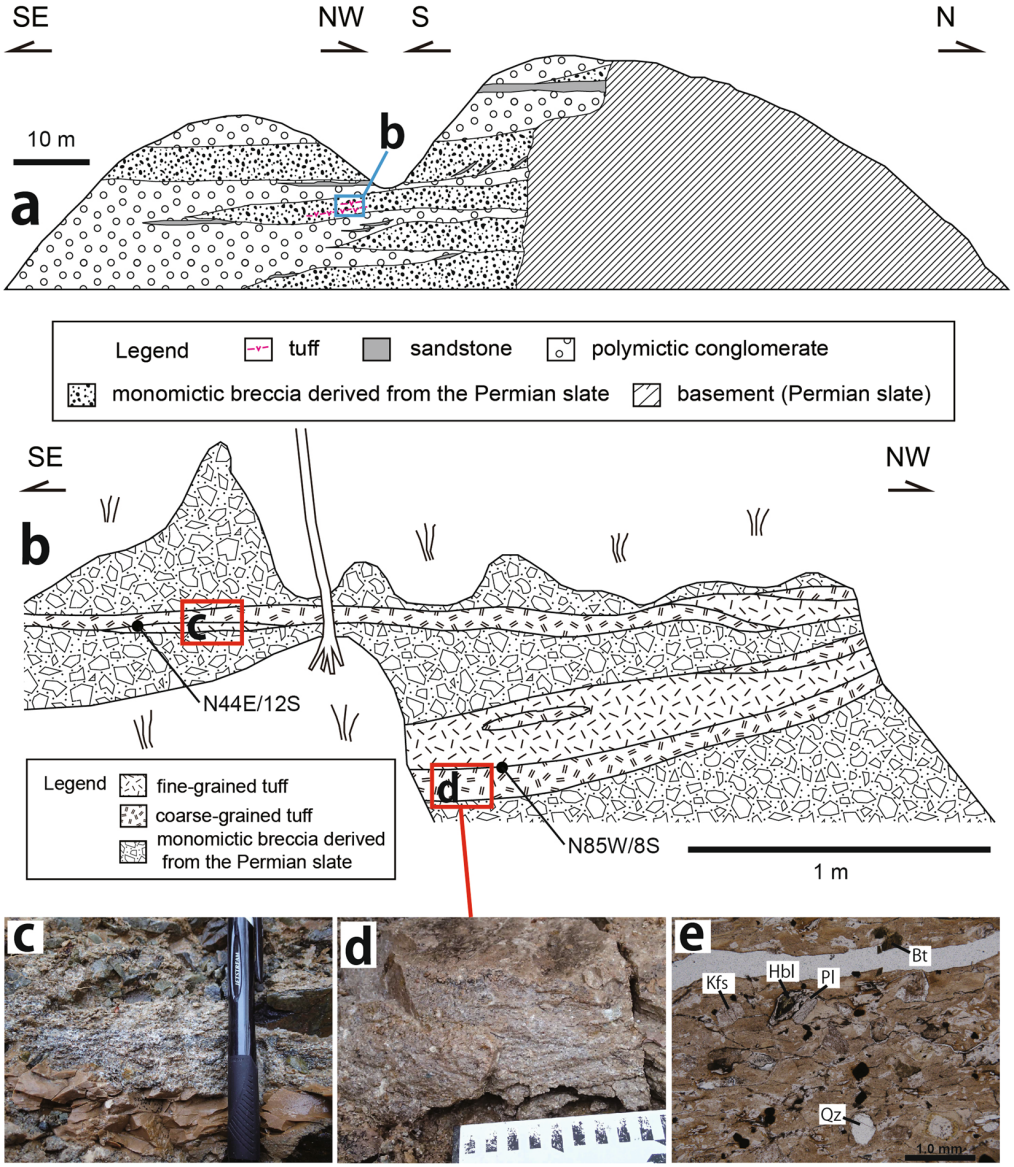
(**a**) Geological sketch of the quadrilateral area marked in Fig. 4a, modified from S. Suzuki’s sketch^4^. (**b**) Sketch showing the occurrence of the tuff in the quadrilateral area (**b**) marked in (**a**). (**c**) Photo of an outcrop consisting of the coarse-grained (greyish white) and the fine-grained (brownish white) tuffs. (**d**) Photo showing the tuff sample collected from the tuff layer intercalated in the conglomerate for LA-ICP-MS U-Pb zircon analyses. The black and white elements of the scale are 1 cm, respectively. (**e**) Photomicrograph of the tuff sample consisting mainly of the brownish volcanic glasses. Hbl: hornblende, Pl: plagioclase, Kfs: K-feldspar, Qz: quartz, Bt: biotite.

**Figure 6:**
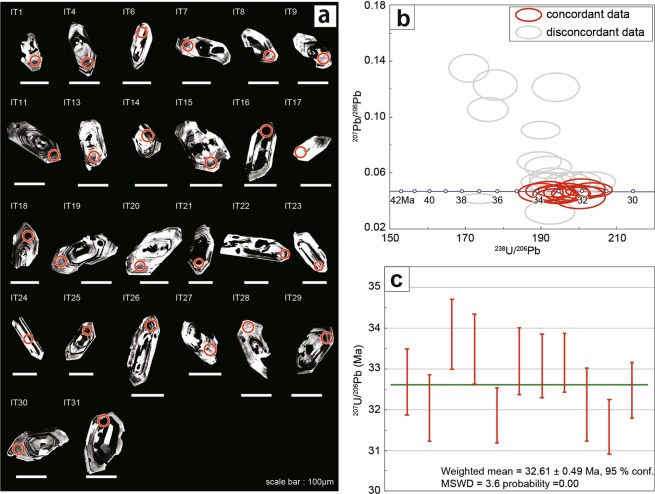
(**a**) Cathodoluminescence images of 31 zircon grains separated from the tuff for LA-ICP-MS U-Pb analyses. Red circles indicate the laser ablation area of the 35-μm diameter. IT1-IT31 are the sample numbers shown in Table 3. (**b**) Tera-Wasserburg diagram (^207^Pb/^206^Pb vs ^238^U/^206^Pb) of the LA-ICP-MS U-Pb analyses of 31 zircon grains separated from the tuff sample (Fig. 5d). (**c**) Weighted mean ^206^Pb/^238^U age (32.61 ± 0.49 Ma) for 11 concordant zircons, ranging from 90% to 110% of the concordance.

The fluvial deposits, including Miocene sediments in the Kibi Plateau region, have been studied by the geomorphologists^2,3^ in relation to the genesis of the peneplain. In the large sport square in Susai town of Akaiwa city, which is situated at the eastern part of the Kibi Plateau (Fig. 4a), S. Suzuki^4^ revealed that the fluvial deposits mainly consist of highly consolidated conglomerates where Eocene tuffs are intercalated. Suzuki named the deposits the Susai Formation of the Kibi Group. The gravels (174 samples larger than 1 cm) in the conglomerates were collected from an area of 0.7 m × 1.4 m (Fig. 4b) to determine their rock types. The gravels are variable in their size, shape (angular to rounded gravel) and rock type (Fig. 4c), being composed of felsic volcanics (47%), intermediate to mafic volcanics (6%), felsic plutonics (5%), intermediate to mafic plutonics (15%), psammites (9%), pelites (16%) and others including chert (2%). The sedimentary rock gravels with angulated shapes originate from the basement rocks (Permian Maizuru Group). Its modal abundance increases considerably at the site with proximity to the Permian Maizuru Group basement. A large and fresh granodiorite gravel (Fig. 4d–f) was collected to carry out K-Ar analyses, giving the ages (88.3 ± 2.0 Ma and 86.5 ± 1.9 Ma) of hornblende and plagioclase, respectively (Table 2). This suggests that the felsic to mafic plutonics in the conglomerates originated from the large-scale igneous activity region, thereby forming a caldera and batholith in the Cretaceous^11,15–17^. The felsic to mafic volcanics in the conglomerates also originated from the same large-scale igneous activity region because the volcanics and plutonics coexist with each other in the Cretaceous igneous activity region^15,16,18^. The tuffs intercalated in the conglomerate gave a zircon Fission Track age of 34.3 ± 1.8 Ma^4^. The two thin (5–10 cm) and thick (ca. 25 cm) tuff layers are observed in the breccia layer close to the basement rocks (Permian Maizuru Group) (Fig. 5). Each layer consists of coarse-grained (greyish white) and fine-grained (brownish white) tuffs (Fig. 5b,c). The sample was collected from the greyish white tuff in the thick layer to carry out LA-ICP-MS U-Pb zircon analyses. The photomicrograph of the collected sample (Fig. 5d) shows that it mainly consists of the brownish volcanic glasses (Fig. 5e). The analytical results (Table 3) of 31 total zircon grains (Fig. 6a) separated from the tuff sample are shown in a Tera-Wasserburg diagram (Fig. 6b). A sample of 11 grains gives concordant ages, with a weighted mean of 32.61 ± 0.49 Ma (Fig. 6c), thereby confirming the Eocene fluvial deposits. This Eocene tuff originated from the large scale felsic igneous activity region, forming the Paleogene cauldrons in SW Japan^11,19,20^.

**Table 2:** K-Ar age data of hornblende and plagioclase separated from the granodiorite sample (Fig. 4d).

Mineral	K content (wt.%)	Rad^40^Ar (10^−8^cc STP/g)	K-Ar age (Ma)	Non-rad^40^Ar (%)
Hornblende	0.471 ± 0.009	165.4 ± 1.8	88.3 ± 2.0	8.7
Plagioclase	1.088 ± 0.022	374.1 ± 4.2	86.5 ± 1.9	10.8

**Table 3:** LA-ICP-MS zircon U–Pb isotopic data. The sample name is the same as the zircon grain number in Fig. 6a.

Sample name	Isotopic ratio						Age (Ma)				Th/U	2σ	Concordance
	207Pb/235U	2σ	206Pb/238U	2σ	207Pb/206Pb	2σ	235U-207Pb	2σ	238U-206Pb	2σ			
IT1	0.02279	0.00507	0.00520	0.00014	0.03179	0.00701	22.9	5.03	33.4	0.92	0.41	0.01	146.1
IT4	0.03742	0.00414	0.00507	0.00013	0.05350	0.00577	37.3	4.05	32.6	0.81	0.54	0.01	87.5
IT6	0.03238	0.00379	0.00508	0.00013	0.04621	0.00529	32.4	3.73	32.7	0.81	0.87	0.01	101.0
IT7	0.03647	0.00384	0.00491	0.00012	0.05385	0.00552	36.4	3.77	31.6	0.78	0.67	0.01	86.9
IT8	0.03339	0.00400	0.00498	0.00013	0.04860	0.00570	33.3	3.93	32.0	0.81	0.63	0.01	96.1
IT9	0.04020	0.00435	0.00518	0.00013	0.05627	0.00591	40.0	4.24	33.3	0.84	0.54	0.01	83.2
IT11	0.03868	0.00384	0.00525	0.00013	0.05343	0.00515	38.5	3.76	33.8	0.81	0.74	0.01	87.6
IT13	0.08627	0.00640	0.00516	0.00013	0.12131	0.00844	84.0	5.99	33.2	0.86	0.62	0.01	39.5
IT14	0.06573	0.00392	0.00527	0.00012	0.09050	0.00502	64.6	3.73	33.9	0.74	0.91	0.02	52.4
IT15	0.04592	0.00479	0.00521	0.00014	0.06394	0.00646	45.6	4.65	33.5	0.87	0.57	0.01	73.5
IT16	0.03312	0.00225	0.00576	0.00012	0.04169	0.00270	33.1	2.21	37.0	0.78	0.93	0.02	111.9
IT17	0.03328	0.00399	0.00526	0.00013	0.04586	0.00537	33.2	3.92	33.8	0.86	0.64	0.02	101.8
IT18	0.09526	0.00741	0.00562	0.00015	0.12289	0.00898	92.4	6.87	36.1	0.96	0.57	0.01	39.1
IT19	0.08252	0.00592	0.00568	0.00014	0.10529	0.00708	80.5	5.55	36.5	0.91	0.82	0.02	45.4
IT20	0.03396	0.00395	0.00521	0.00013	0.04729	0.00537	33.9	3.88	33.5	0.85	0.72	0.02	98.8
IT21	0.03199	0.00211	0.00495	0.00011	0.04682	0.00292	32.0	2.07	31.9	0.68	1.84	0.04	99.7
IT22	0.04954	0.00443	0.00528	0.00013	0.06810	0.00584	49.1	4.28	33.9	0.85	0.70	0.02	69.1
IT23	0.03774	0.00362	0.00500	0.00012	0.05469	0.00507	37.6	3.54	32.2	0.78	0.98	0.02	85.6
IT24	0.03195	0.00359	0.00516	0.00013	0.04488	0.00492	31.9	3.53	33.2	0.82	0.96	0.02	104.0
IT25	0.03077	0.00314	0.00514	0.00012	0.04339	0.00431	30.8	3.10	33.1	0.79	0.88	0.02	107.5
IT26	0.03320	0.00235	0.00516	0.00011	0.04670	0.00315	33.2	2.31	33.1	0.72	1.24	0.03	100.0
IT27	0.02947	0.00477	0.00500	0.00014	0.04279	0.00682	29.5	4.70	32.1	0.89	0.67	0.02	108.9
IT28	0.10883	0.00713	0.00585	0.00015	0.13492	0.00816	104.9	6.53	37.6	0.94	0.73	0.02	35.8
IT29	0.03236	0.00291	0.00491	0.00010	0.04779	0.00418	32.3	2.87	31.6	0.67	0.72	0.02	97.7
IT30	0.03768	0.00446	0.00519	0.00013	0.05270	0.00610	37.6	4.36	33.3	0.81	0.68	0.02	88.8
IT31	0.03145	0.00285	0.00505	0.00011	0.04515	0.00398	31.4	2.81	32.5	0.68	0.84	0.02	103.3

**Figure 7:**
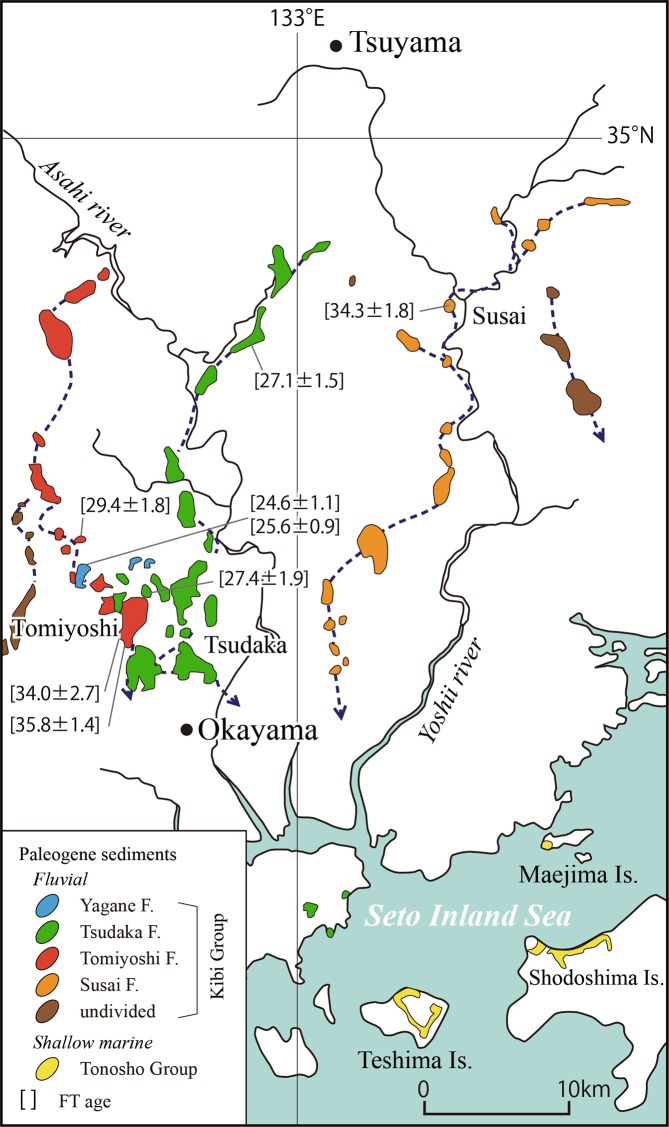
Map showing the distribution of the Kibi Group (Paleogene fluvial deposits) in the eastern part of the Kibi Plateau compiled from the literature^4–6,28^. The numerals in the brackets show the Fission Track ages. The distribution of Paleogene shallow marine sediments without any significant inclination in the Maejima, Teshima and Shodoshima islands in the Seto Inland Sea is also shown.

The deposits were traced for 40 km from north to south and the paleo flow direction was from north to south by boulder imbrication (Fig. 7). This distribution pattern of the Eocene fluvial deposits makes it possible to reconstruct the paleo “Susai” river that flowed in the eastern Asian continent before the Japan Sea opening in Middle Miocene^7,8^. The boundary between the deposits and the basement wall rock (Permian Maizuru Group) was found in the developed land of Susai town (Fig. 5a), which suggested that the paleo “Susai” river was wider than 300 m there. The width may have reached at least 1 km in the south as deduced from the largest deposit distribution. Areal mapping and zircon Fission Track analyses of the tuffs in the eastern area of the Kibi Plateau region have revealed three paleo rivers, where the deposits can be divided into two groups with the ages of 27–29 Ma (Oligocene) and 34–36 Ma (Late Eocene) (Fig. 7). The former deposits are called the Tsudaka Formation and the later deposits are the Susai and Tomiyoshi Formations of the Kibi Group. The Susai Formation, which belongs to the Late Eocene group, is observed at the elevation (93–119 m) in the developed land mentioned previously (Fig. 4a). This elevation is 50–76 m higher than the present Yoshii river (43 m) ca. 500 m NE of the land.

**Figure 8:**
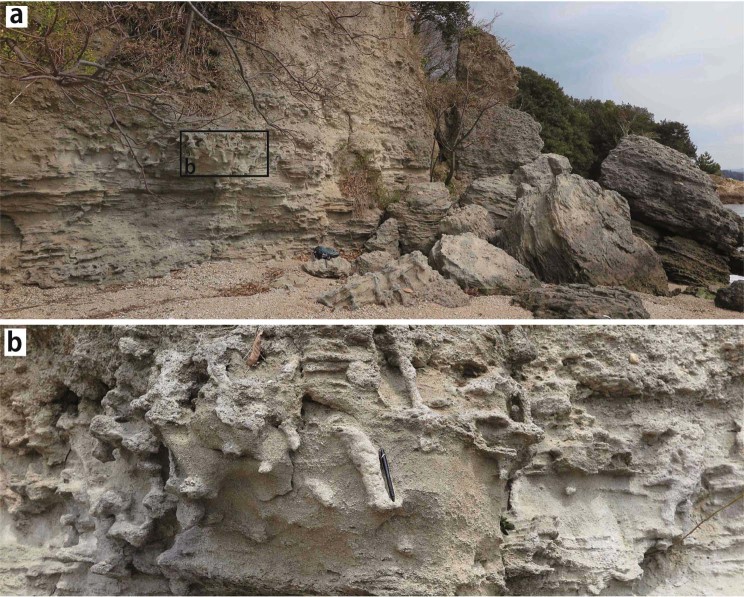
(**a**) Photo of the outcrop of Paleogene shallow marine sediments without any significant inclination observed in the small island (Maejima) of Seto Inland Sea. The sediments consist mainly of sandstone and conglomerates containing the fossils of *Molluscan fauna*^22^ and *Scalpellid-dominated barnacle assemblages*^23^, and trace fossils^21^. The rectangular box shows the area of the photo (**b**). (**b**) Photo of the rectangular part (**b**) in photo (**a**) showing the trace fossils.

Paleogene to Miocene shallow marine sediments without any significant inclination (Fig. 8a) have been observed in the small island (Maejima) of the Seto Inland Sea^21–23^. The sediments unconformably overlie the Cretaceous granitic rocks, with a magma mingling structure^21^ and consisting mainly of sandstone and conglomerates containing the fossils of *Molluscan fauna*^22^, *Scalpellid-dominated barnacle assemblages*^23^ and trace fossils^21^ (Fig. 8b). The Eocene shallow marine sediments containing the fossils of *Dinoflagellate cyst assemblages* without any significant inclination are also observed in the Tonosho Group of Shodoshima and Teshima south of Maejima^24,25^ (Fig. 7). This finding suggests that these shallow marine sediments might have formed in the paleo-Pacific Ocean coast area of the eastern Asian continent in Eocene. Middle Miocene shallow marine sediments without any significant inclination were also observed in an extremely small area within the Kibi Plateau region. The deposits contain many fossils, such as *Vicarya-Anadara* and *Geloina* assemblages^26^, and are correlated with the marine sediments of the Katsuta Group^27^ occurring in the north east part of the Kibi Plateau. The tuffs intercalated in these deposits give Fission Track zircon ages of 16 Ma for the former and 16 and 18 Ma for the latter^4,6^. These geological data for the sediments without any significant inclination and the reconstructed paleo-rivers without any significant displacement suggest that the Kibi Plateau region, including the shallow marine sediments distribution area, was stable since at least late Eocene (ca. 34 Ma) through the Japan Sea opening and the associated quick rotation (an angular velocity of 20°/Myr) of SW Japan in the Middle Miocene^7,8^. These Paleogene fluvial deposits are sporadically and widely distributed in the Kibi Plateau region^5,28^. Their total distribution area, including the shallow marine sediments mentioned previously, is ca. four times larger than the area of Tokyo (1,787 km^2^) on the main island without its minor islands (407 km^2^).

## Discussion

Seismic tomography of the crust and upper mantle beneath SW Japan^9,10^ shows that the Kibi Plateau area exhibits significantly high velocities (i.e., high-Vp and high-Vs) and low attenuations (i.e., high-Qp and high-Qs). The high-V and high-Q anomalies in the Kibi Plateau area are visible in the upper crust down to an approximate 20 km depth. This high-V and high-Q anomaly zone completely covers the Paleogene fluvial deposit distribution area known as the Kibi Plateau region. No active faults have been observed^29^ in the central part of the Kibi Plateau, although faults with a Permian to Cretaceous lithology are observed on the geological map^11^. The seismic activity is low within the Kibi Plateau situated in the main central part of the high-V and high-Q anomaly zone, whereas crustal earthquakes occur actively in the low-V and low-Q zones surrounding the Kibi Plateau^9,10^. These seismological results support that the Kibi Plateau region will be a stable-coherent tectonic unit in the future.

Thus, the geological and geophysical data described in this study indicate that the Kibi Plateau region has behaved as a stable-coherent tectonic unit (at least 7,000 km^2^ × 20 km = 140,000 km^3^) since the Eocene and will continue to be stable in the future. This behavior is highly interesting to researchers living in the active Japanese Islands. Why and how this size of a stable- and rigid-coherent tectonic unit formed in the Eocene and has been preserved up to the present? We will propose the following possible story to explain the reason. The Kibi Plateau region that has now a thick crust over 30 km^30^ existed as a stable eastern segment of the Asian continent in the Eocene when the large scale felsic igneous activity to form the Paleogene cauldrons took place in the area north of the Kibi Plateau region^20^. During the Japan sea opening from early to Middle Miocene^7^, SW Japan including the Kibi Plateau tectonic unit drifted to the south with a velocity of 14 cm/y as estimated from ref. ^8^. At the time, the Philippine Sea plate subduction was forced to cause the Middle Miocene Setouchi volcanism to form the high Mg andesite magma^31^ and the Median Tectonic Line formed in the Cretaceous between the inner (including the Kibi Plateau region) and outer zones of SW Japan was reactivated to form the structure that the inner zone thrusted over the outer zone^32^. These successive tectonic events protected the Kibi Plateau tectonic unit from the destruction. The Kibi Plateau region has not experienced any subduction related arc volcanism since the Eocene. This has also influenced to preserve the stable-coherent tectonic unit.

As described previously, the Susai Formation that appeared on the developed land exists at an elevation (93–119 m) that is 50–76 m higher than the present Yoshii river (43 m). This means the average uplift level from the Late Eocene (34 Ma) is 50–76 m if the paleo-Susai river flowed at the same level as the present Yoshii river, thereby showing an average uplift rate of 50–76 m/34 Ma (=0.0015–0.0022 mm/y). This value is much lower than the values (3.4–6.1 mm/y) observed in the Kiso Range, central Japan^33^. This extremely slow uplift is consistent with the red soil formation observed at the low-relief surface in the Kibi Plateau. We can now confirm the existence of a stable-coherent tectonic unit (at least 140,000 km^3^) in the active Japanese Islands. Since Tokyo as a capital city is highly vulnerable to natural disaster, this stable-coherent tectonic unit should be used effectively by the Japanese people, e.g., as their capital city and/or as a relocation area for very important capital functions. This approach for revealing the stable-coherent tectonic unit in the active Japanese Islands will be applicable to the active islands with marginal sea like Japan sea in the world, e.g., in the Balearic Islands and Corsica-Sardinia where the rotational motion associated with opening of marginal seas are observed^34,35^.

## Methods

No statistical methods were used to predetermine the sample size. The experiments were not randomized. The investigators were not blinded to allocation during the experiments and outcome assessment.

### Definition of the U-shaped valley

The valleys all have the form of a “U” or “V”. A U-shaped valley is a geological formation characterized by high and steep sides and a rounded or flat valley bottom. The cross-section of the U-shaped valley is expressed by the form Y = a X^b^, where b ranges between 1.5 and 2.1^36^. We encountered U-shaped valleys in the Kibi Plateau (Fig. 1c,d). The form of the “U” is thoroughly approximated by a parabola (Y = a X^2^).

### Whole-rock major analyses

Whole-rock major analyses of the deeply weathered rocks and their host rocks were carried out to observe the chemical weathering process. The samples were collected from the four sites: pelite (RS1) and the schistose rocks (RS4) from the Permian Maizuru Group, as well as Cretaceous rhyolite (RS2) and Cretaceous granites (RS3) in the northern part of Akaiwa city (Fig. 3a). The sample (RS5) was also collected from the red soil exploitation site (Fig. 2a: the host is Cretaceous granite) for use as an impermeable material for the local farm pond. The red soil samples to be analysed were collected from the layer far from the surface to avoid (as much as possible) the effect of the plant roots. Major elemental contents of the red soil and the host rock samples were determined at the Okayama University, Japan, using a Rigaku ZSX Primus II XRF spectrometer following the procedures of ref. ^37^. The red soil samples were dried at room temperature condition for three days, and then, ca. 10 g of the dried samples were pulverized in an agate mortar. The host rock samples occurring as a core stone were collected to avoid (as much as possible) weathered surfaces and veins. The samples were crushed manually in an iron mortar and then pulverized in an agate mortar. Both types of powder samples were heated at 800 °C for 10 hours to determine the loss of ignition using the gravimetric method. Glass beads were prepared by mixing 1.8 g of the sample powder and 3.6 g of a mixed alkali flux (Li_2_B_4_O_7_: LiBO_2_ = 4:1) before fusion in an automated bead sampler. To check the analytical precision and accuracy, three standard samples of the Geological Survey of Japan (JG-1a, JA-1 and JB-1a) were analysed. The standard deviations for all elements are less than 0.1, and the values are in accordance with previously reported reference values^38^. The host rocks of the weathered schistose rock (RS4) and pelite (RS1) from Permian Maizuru Group were not analysed because their fresh samples could not be collected near their weathered rocks. The results are shown in Table 1.

### K–Ar dating

A large granodiorite gravel (Fig. 4d) was collected to date the minerals in the gravel from the sampling site (d) shown in Fig. 4a. Photomicrographs (Fig. 4e,f) of the granodiorite show that the rock consists mainly of hornblende, plagioclase, K-feldspar and quartz, and accessory apatite and opaque. The granodiorite sample was crushed in a jaw crusher and sieved for mineral separation. The sieved fraction was washed in distilled water in an ultrasonic bath to remove any fine particles on the grain surfaces and then dried in an oven at 80 °C. Magnetite was removed with a hand magnet. The magnetite-free fraction was then passed through an isodynamic magnetic separator to separate hornblende and plagioclase. The separated minerals were used for argon analysis. Separate aliquots were further pulverized in an agate mortar for potassium analysis. Potassium concentrations in the hornblende and plagioclase fractions were determined by flame photometry using the method from a previous study^39^. Argon was analysed at Okayama University of Science using a 15 cm radius sector type mass spectrometer with a single-collector, utilizing isotope dilution and argon-38 spike methods^40^. Mass discrimination was checked with atmospheric argon each day. Specimens wrapped in Al foil were vacuumed out at 150–200 °C for approximately 24 hours, and argon was then extracted at 1500 °C in an ultra-high vacuum line. Reactive gases were removed using a Ti– Zr scrubber. The decay constants for ^40^K to ^40^Ar and ^40^Ca, and ^40^K contents in potassium used in the age calculation are 0.581 × 10^−10^/y, 4.962 × 10^−10^/y and 0.0001167, respectively^41^. The results are shown in Table 2.

### Cathodoluminescence analyses of zircon

The zircon grains for LA-ICP-MS U-Pb dating were separated using the conventional sieving, magnetic, and heavy liquid techniques, and then mounted on epoxy disks. Their internal structures were observed with a XM-26740PCLI cathodoluminescence (CL) spectrometer coupled to a JXA-8230 electron probe microanalyzer (JEOL Ltd., Tokyo, Japan) at the Okayama University of Science (OUS) before isotopic analysis (Fig. 6a).

### LA-ICP-MS U-Pb zircon dating

*In situ* U–Pb dating of zircon grains was undertaken at the Okayama University of Science by using a Thermo Scientific iCAP-RQ single-collector quadrupole ICP-MS coupled to a Teledyne Cetac Technologies Analyte G2 laser ablation (LA) system that utilizes a 193-nm ArF excimer laser. The zircon mount was set in a two-volume HelEx II 2-Volume sample cell of the LA system. The areas free of cracks and inclusions in each zircon were chosen for the analysis with an LA camera. Before the analysis, the analytical areas were pre-ablated using a laser pulse of 50-μm diameter for removing potential surface contaminants on the zircon surfaces. After shooting the laser with the shutter closed for 30 s (with the laser warming up), the areas were ablated with a laser of 35-μm diameter with a fluence of 1.58 J/cm^2^ and a repetition rate of 5 Hz. During the analysis, He gas was introduced into a HelEx II 2-Volume sample chamber (MFC1) and the HelEx II arm (MFC2) as a carrier gas. The flow rates into MFC1 and MFC2 were set to 0.5 L/min and 0.3 L/min, respectively. The ablated sample aerosol in He gas was carried through the signal-smoothing device “squid” to the ICP-MS. Before the analysis, the ICP-MS was optimized by using continuous ablation of a NIST SRM 612 glass standard to provide maximum sensitivity of ^238^U and ^206^Pb while maintaining low oxide formation (^232^Th^16^O/^232^Th <1%). A total of 6 nuclides (^202^Hg, ^204^Pb, ^206^Pb, ^207^Pb, ^232^Th and ^238^U) were analysed with the ICP-MS. The background and ablation data for each analysis were collected for 15 s of the laser warming-up time and 15 s of the ablation time, respectively. The background intensities were subtracted from following the signals at the ablations. Those data were acquired for multiple groups of 15 unknown grains bracketed by a trio of analyses of the 91500 zircon standard^42,43^ and NIST SRM612 glass standard, which were used for corrections of the ^206^Pb/^238^U and ^207^Pb/^206^Pb ratios, respectively. As a normalization value of the 91500 zircon standard, an apparent ^206^Pb/^238^U without common Pb correction was used^44^ (i.e., ^206^Pb/^238^U = 0.17928 ± 0.00018). The instrumental mass bias of ^207^Pb/^206^Pb ratios was corrected by normalizing to compiled values of the NIST SRM612 glass standard^45^. All uncertainties are quoted at a 2-sigma level in which the repeatability of each six measurements of the 91500 zircon and NIST SRM612 data bracketing unknown sample groups is propagated. Elemental fractionation of the U/Pb and Th/U ratios and mass fractionation of the ^207^Pb/^206^Pb ratio were linearly interpolated by the measured data of the six analyses of 91500 zircon and NIST SRM612, respectively. ^235^U intensities were calculated from ^238^U using a ^238^U/^235^U ratio of 137.88^46^. Through all the analyses, Plešovice zircon^47^ was measured as a secondary standard for quality control. The weighted mean ^206^Pb/^238^U ages of 336.1 ± 3.3 Ma and ^207^Pb/^235^U age of 332.4 ± 5.5 Ma were obtained at the laser spot size diameter of 35 μm (n = 3). Those values are coincident with the reference age of 337.13 ± 0.37 Ma^47^. The analyses of thirty-one grains were conducted. Each result is shown in Table 3. Their Th/U ratios are higher than 0.1. The concordance was defined as the value of 100% × (^206^Pb/^238^U age)/(^207^Pb/^235^U age).
